# Sensitive detection of PD-L1 expression on circulating epithelial tumor cells (CETCs) could be a potential biomarker to select patients for treatment with PD-1/PD-L1 inhibitors in early and metastatic solid tumors

**DOI:** 10.18632/oncotarget.20346

**Published:** 2017-08-18

**Authors:** Dorothea Sonja Schott, Monika Pizon, Ulrich Pachmann, Katharina Pachmann

**Affiliations:** ^1^ Transfusion Center Bayreuth, Bayreuth, Germany

**Keywords:** circulating epithelial tumor cells, programmed cell death ligand 1, programmed cell death ligand 2, checkpoint inhibitors

## Abstract

**Background:**

The current cancer research strongly focuses on immune therapies, where the PD-1, with its ligands plays an important role. It is known that PD-L1 is frequently up-regulated in a number of different cancers and the relevance of this pathway has been extensively studied and therapeutic approaches targeting PD-1 and PD-L1 have been developed. We used a non-invasive, real-time biopsy for determining PD-L1 and PD-L2 expression in CETCs of solid cancer patients.

**Methods:**

CETCs were determined from blood of 128 patients suffering from breast (72), prostate (27), colorectal (18) and lung (11) cancer. The number of vital CETCs and the expression of PD-L1 and PD-L2 were investigated using the maintrac^®^ method.

**Results:**

PD-L1 expressing CETCs were detected in 94.5% of breast, 100% of prostate, 95.4% of colorectal and 82% of lung cancer patients whereas only 75% of breast cancer patients had PD-L2 positive CETCs. In the PD-L1 and PD-L2 expressing patients the cell fraction of PD-L1 positive CETCs is significantly higher than the fraction of PD-L2 positive CETCs (54.6% vs. 28.7%; p<0.001). Breast cancer patients with metastatic disease had significantly more PD-L1 positive CETCs as compared to patients without metastasis (median 75% vs. 61.1%; p<0.05).

**Conclusion:**

PD-L1 seems to be a major factor in immune evasion and is highly expressed on CETCs regardless of the type of cancer. Monitoring the frequency of PD-L1 positive CETCs could reflect individual patient's response for an anti-PD-1/PD-L1 therapy and may be a promising target of anticancer treatment.

## INTRODUCTION

Metastatic disease is responsible for over 90% of cancer-related deaths. To date the characteristics of the primary tumor are used to predict the probability of tumor progression and metastatic relapse. However, the clonal landscape of the overall tumor burden is very heterogeneous and a single biopsy may fail to represent the whole cancer cell population. Furthermore, the biopsy is invasive and repeating the procedure is not always feasible due to safety concerns [1, 2].

Most of metastases are due to hematogenous dissemination of tumor cells from the primary tumor which starts at an early stage of cancer growth. Single tumor cells or cell clusters shed from the primary tumor travel to distant organ sites and can grow into metastatic lesions [3]. Aggressive tumors may release thousands of cancer cells into the circulation but only a small part of them can survive and <0.01% eventually succeed in forming metastasis [2]. The detection of circulating tumor cells presents a technical challenge, because these cells are assumed to be rare. Most studies performed in the metastatic situation have shown a significant correlation between overall survival and the number of circulating tumor cells detectable with the respective approaches [4]. Using a nondissipative method avoiding cell loss [5] circulating tumor cell counts can be used as a marker for therapy response also in the adjuvant situation allowing continuous monitoring during treatment [6]. These cells can not only be followed over time but also further characterized at any time during the course of disease.

Therefore, using circulating tumor cells as a “liquid biopsy” holds great potential to better represent the actual composition of tumor cells with minimal risk for patients. It can be repeated frequently for real-time monitoring of cancer treatment and can give important information on therapeutic targets and drug resistance mechanisms [1, 2, 7, 8, 9].

In order to successfully evade immune surveillance, tumor cells use a variety of different strategies. One of them is the upregulation of surface programmed cell death ligand 1 (PD-L1, CD247, B7-H1) expression [10]. PD-L1 is a 40 kDa transmembrane protein that is expressed on activated immune cell types including natural killer cells, macrophages, myeloid dendritic cells, B cells, and vascular endothelial cells as well as numerous epithelial cells including cancers. The physiologic role of PD-L1 is to bind to the programmed cell death 1 receptor (PD-1) expressed on the surface of activated cytotoxic T cells [10, 11]. The PD-1/PD-L1 interaction serves as an important regulatory checkpoint against an excessive adoptive immune response to antigens and autoimmunity [10]. This binding causes inhibition of IL-2 production and T cell activation. The second ligand for PD-1 is PD-L2 (also known as B7-DC and CD273) but its role in modulating immune responses is less clear and only little information is available. Generally, PD-L2 is expressed at a lower level than PD-L1 but the relative affinity of PD-L2 to PD-1 is 2-6-fold higher than that of PD-L1 [12]. The expression of PD-L1 has been evaluated in a number of tumor types in different localizations like head and neck, lung, stomach, colon, pancreas, breast, kidney, bladder, ovary, cervix, as well as melanoma, glioblastoma, multipole myeloma, lymphoma, and various leukemias [11]. Although most of the analyses of PD ligand expression have focused on PD-L1 PD-L2 has also been reported to be upregulated in various tumors with distinct expression profiles such as certain B cell lymphomas and Hodgkin's disease [13, 14]. Anti-PD-1 and anti-PD-L1 drugs which should restore anti-cancer immunity have been developed and are now available for clinical use. Numerous novel checkpoint-inhibitors are being tested now in clinical trials. Durable responses have been observed in different cancers including melanoma, renal, lung, prostate, and bladder carcinomas [15, 16].

To date, however, there is no reliable predictive biomarker for determining the response rate for a targeted PD-1/PD-L1 therapy. It has been shown that PD-L1 expression by tumor and/or infiltrating immune cells correlates with a therapeutic response [17]. The intra- tumor heterogeneity observed in both primary and metastatic sites, with significantly higher PD-L1 expression in metastatic sites, indicates that a single core biopsy might not be sufficient to determine PD-L1 expression. For this reason the primary tumor may not be an adequate surrogate for determining PD-L1 expression in metastatic sites [18]. These distant sites of disease represent aggressive subclones that were able to disseminate from the primary tumor and to escape immune destruction, therefore identifying PD-L1 on circulating tumor cells could be a new biomarker for better selecting patients for treatment with PD-1/PD-L1 antibodies [10, 18]. Because cells that are able to effectively evade cytotoxic T cells would have a greater selective advantage and likely contribute more to the progression of cancer disease. Effective immune eradication of these highly invasive cells through PD-L1 antibody therapy may be an effective strategy for arresting the progression of cancer [10]. The purpose of our study was to better characterize PD-L1/−L2 expression on circulating epithelial tumor cells (CETCs) in solid tumors which might contribute a new biomarker for targeted PD-1 and PD-L1 therapy.

## RESULTS

**Figure 1:**
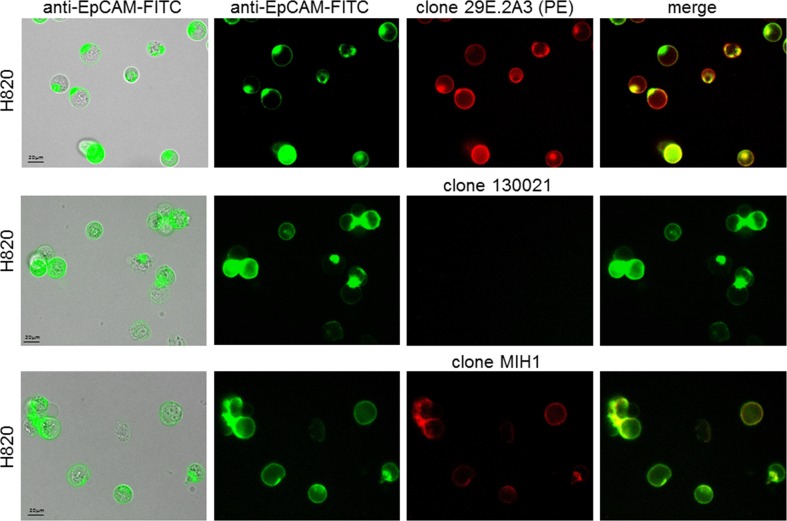
Specificity of different anti-human PD-L1 antibodies H820 cell line was analyzed using three different anti-human PD-L1 MAb-PE clones: clone 29E.2A3, clone 130021, clone MIH1. The cells from the cell line H820 were positive for EpCAM (green) and PD-L1 (red) with clone 29E.2A3 and clone MIH1 and negative with clone 130021

**Figure 2:**
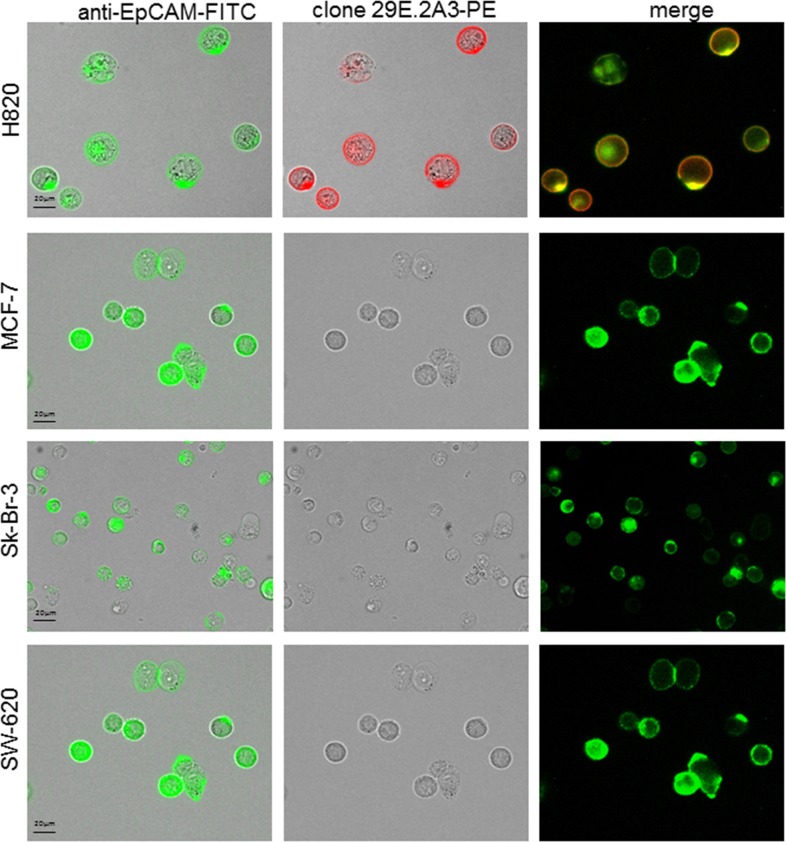
Specificity of clone 29E.2A3 with four different cell lines H820 cells were positive for PD-L1 staining, whereas MCF-7, Sk-Br-3 and SW620 cells were completely negative.

For the development of an approach to detect PD-L1 or PD-L2 on CETCs the specificity of antibodies was determined. Therefore we analysed different cancer cell lines H820, Sk-Br-3, MCF-7 and SW-620 by using fluorescence scanning microscope. H820 was strongly positive for PD-L1 only with clones 29E.2A3 and MIH1 and completely negative with clone 130021 (Figure 1). The specificity of the staining was demonstrated by the fact that no signals were detected for the SW-620, MCF-7 and Sk-Br-3 cell lines or with isotype control antibody (Figure 2). To further evaluate to specificity of the antibody, Sk-Br-3 was treated with IFN-γ. However, in our hands, PD-L1 expression in Sk-Br-3 cells remained unchanged. Clone 29E.2A3 was selected for further investigations because lack of unspecific staining.

**Figure 3:**
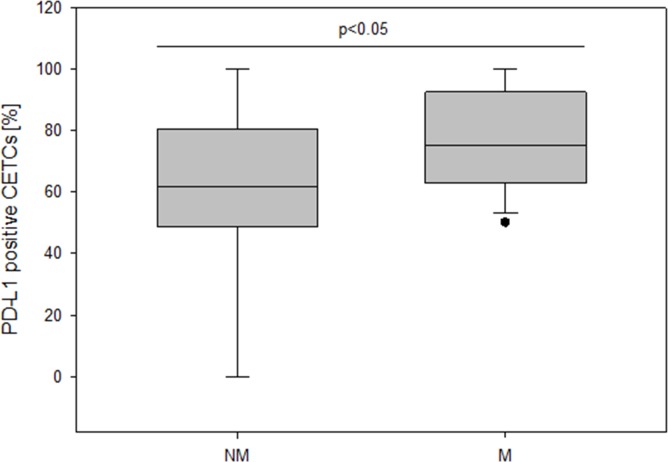
The frequency of PD-L1 positive CETCs (%) in non-metastatic and metastatic breast cancer patients

The patient characteristics according to PD-L1 expression are shown in Table 1. The median number of CETCs was 55/100μl blood (ranging from 5 to 805), 65/100μl blood (ranging from 5 to 905), 55/100μl blood (ranging from 5 to 650) and 40/100μl blood (ranging from 5 to 95) in breast, prostate, colorectal and lung cancer patients, respectively. As negative control we tested blood samples from 25 healthy controls and confirmed that none of the samples were positive for CETCs. We found no statistically significant difference between the number of CETCs as well as PD-L1 positive CETCs and tumor entities. PD-L1 positive CETCs were observed in 68 breast (94.5%), 27 prostate (100%), 17 colorectal (94.5%) and 9 lung (82%) cases. Median percentage of PD-L1 positive cells among the CETCs was 68.9 (range: 0-100) in breast, 65.8 (range: 32-100) in prostate, 57 (range: 0-90.5) in colorectal and 55 (range 0-90) in lung cancer. The absolute number of PD-L1 positive CETCs did not correlate with any clinicopathological parameters except with the presence of distant metastasis and radiotherapy in breast cancer patients. Patients with metastatic disease exhibited a significantly higher fraction of PD-L1 positive CETCs as compared to patients without metastasis (median 75% vs 61.1%; p<0.05) (Figure 3).

**Table 1:** Characteristics of cancer patients and control group according to PD-L1 expression. (FEC=5-fluorouracil, epirubicin, cyclophosphamide; EC= epirubicin, cyclophosphamide; TAC= docetaxel, doxorubicin, cyclophosphamide; HF=hypofractionated radiation therapy)

Clinicopathological Parameters	PD-L1 positive CETCs	PD-L1 negative CETCs	Median of PD-L1 positive CETCs (%)	p value
**Breast cancer**				
**Gender**				
Female	68 (94.5%)	4 (5.5%)		
Male				
**Age**				p>0.05
<50 years	19 (90.5%)	2 (9.5%)	70	
>50 years	49 (96%)	2 (4%)	67.9	
**Tumor size**				p>0.05
T1	38 (97.4%)	1 (2.6%)	75	
T2	13 (86.6%)	2 (13.4%)	64.3	
T3	7 (87.5)	1 (12.5%)	69.9	
T4				
n.a. (n=10)				
**Lymph node metastasis**				p>0.05
Positive	28 (96.5%)	1 (3.5%)	70	
Negative	25 (96%)	1 (4%)	76	
n.a. (n=17)				
**Distant metastasis**				p<0.05
Positive	17 (100%)	0 (0%)	75	
Negative	48 (88.9%)	6 (11.2%)	61.7	
n.a. (n=1)				
**HER2 status**				p>0.05
Positive	8 (88.9%)	1 (11.1%)	67.9	
Negative	42 (97.7%)	1 (2.3%)	70	
n.a. (n=20)				
**ER status**				p>0.05
Positive	53 (94.6%)	3 (5.4%)	73	
Negative	12 (100%)	0 (0%)	70	
n.a. (n=4)				
**Chemotherapy**				p>0.05
Adjuvant	38 (97.4%)	1 (2.6%)	71.4	
• FEC	19 (95%)	1 (5%)	76.6	
• EC	11 (100%)	0 (0%)	56.9	
• TAC	8 (100%)	0 (0%)	83.4	
Neoadjuvant (EC)	4 (100%)	0 (0%)	70	
No	27 (93%)	2 (7%)		
**Endocrine therapy**				p>0.05
Yes	44 (100%)	0 (0%)	72	
No	23 (92%)	2 (8%)	68.1	
n.a = 3				
**Radiation**				p<0.05
Yes	30 (100%)	0 (0%)	77.4	
• HF	10 (100%)	0 (0%)	79.5	
• Standard	20 (100%)	0 (0%)	70.7	
No	31 (100%)	0 (0%)	62.5	
n.a. (n=11)				

Clinicopathological Parameters	PD-L1 positive CETCs	PD-L1 negative CETCs	Median of PD-L1 positive CETCs (%)
**Prostate cancer**			
**Age**			
<60 years	6 (100%)	0 (0%)	61
>60 years	21 (100%)	0 (0%)	73.4
**Stage**			
I	3 (100%)	0 (0%)	75
II	4 (100%)	0 (0%)	72.1
III	4 (100%)	0 (0%)	42.6
IV	12 (100%)	0 (0%)	68.1
n.a. (n=4)			
**Lymph node metastasis**			
Positive	8 (100%)	0 (0%)	68.8
Negative	9 (100%)	0 (0%)	81.1
n.a. (n=10)			
**Distant metastasis**			
Positive	15 (100%)	0 (0%)	65
Negative	11 (100%)	0 (0%)	66.7
n.a. (n=1)			
**Chemotherapy**			
Yes	6 (100%)	0 (0%)	71.8
No	15 (100%)	0 (0%)	69.5
n.a (n=6)			
**Radiation**			
Yes	6 (100%)	0 (0%)	70.25
No	17 (100%)	0 (0%)	66.7
n.a. (n=4)			
**Colorectal cancer**			
**Gender**			
Female	9 (100%)	0 (0%)	60
Male	8 (89%)	1 (11%)	55
**Age**			°
<60 years	6 (86%)	1 (14%)	60
>60 years	11 (100%)	0 (0%)	56
**Stage**			
I	1 (100%)	0 (0%)	56
II	2 (100%)	0 (0%)	46.5
III	3 (100%)	0 (0%)	55
IV	9 (90%)	1 (10%)	63.3
n.a. (n=2)			
**Lymph node metastasis**			
Positive	10 (91%)	1 (9%)	66.6
Negative	5 (100%)	0 (0%)	56
n.a. (n=2)			
**Distant metastasis**			
Positive	9 (90%)	1 (10%)	15
Negative	8 (0%)	0 (0%)	12.5
**Chemotherapy**			
Yes (FOLFOX)	10 (100%)	0 (0%)	66
n.a. (n=1)			

Clinicopathological Parameters	PD-L1 positive CETCs	PD-L1 negative CETCs	Median of PD-L1 positive CETCs (%)
**Lung cancer**			
**Gender**			
Female	2 (67%)	1 (33%)	56.6
Male	7 (88%)	1 (12%)	55.5
**Age**			
<60 years	1 (33%)	2 (67%)	44.6
>60 years	8 (100%)	0 (0%)	66.8
**Stage**			
I	0 (0%)	1 (100%)	0
II	1 (100%)	0 (0%)	55
III	2 (100%)	0 (0%)	41.6
IV	5 (83%)	1 (17%)	78.6
n.a. (n=1)			
**Distant metastasis**			
Positive	4 (80%)	1 (20%)	78.6
Negative	4 (66.7)	2 (33.3%)	47.8
**Chemotherapy**			
Yes	2 (100%)	0 (0%)	64.3
No	4 (57%)	3 (43%)	33.3
n.a. (n=2)			
**Control group**			
**Gender**			
Female	0 (0%)	10 (100%)	
Male	0 (0%)	15(100%)	
**Age**			
<30 years	0 (0%)	8 (100%)	
>30 years	0 (0%)	17 (100%)	

**Figure 4:**
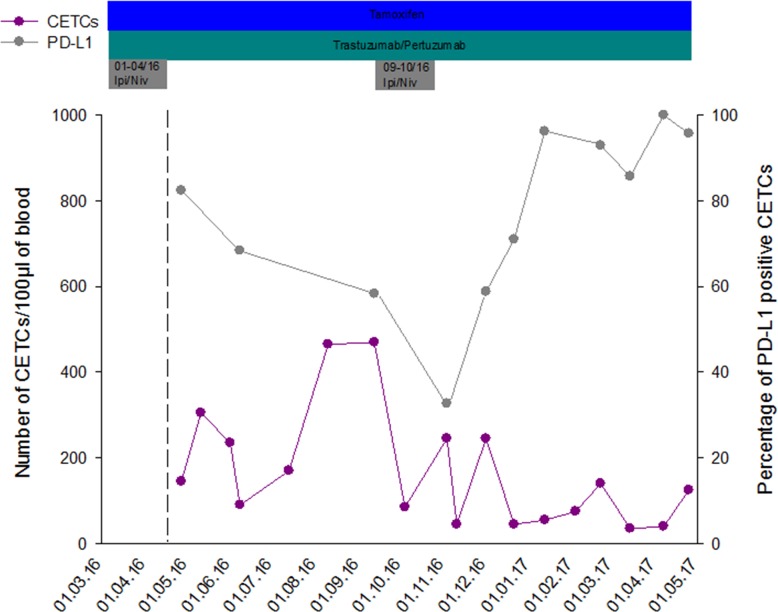
Exemplary course of number of CETCs and frequency of PD-L1 positive CETCs in one primary metastatic breast cancer patient during combined therapy with Nivolumab (Niv) and Ipilimumab (Ipi)

Figure 4 shows a representative serial analysis of CETCs and PD-L1 expression in one exemplary primary metastatic breast cancer patient (cT2 cN2 M1 (liver); ER: +, PR: +, HER2/neu: 3+, Ki-67: 30%) who had received chemotherapy and now is under antibody and hormone therapy. She was treated with a combination of Ipilimumab and Nivolumab. After first administration of immune-checkpoint inhibitors (01-04/16) the remnant metastases in the liver were significantly reduced and the number of CETCs initially was at a very low level. The proportion of PD-L1 positive cells was above 80%. CETC numbers first decreased but subsequently we observed an increase in cell numbers. The patient then received the second dose of immunotherapy (09-10/16) leading to a sharp decrease in CETC numbers as well as the frequency of PD-L1 expression on these cells. During the following 6 months of follow-up without immunotherapy the number of CETCs persists at a low level but the percentage of PD-L1 positive CETCs increases continuously achieving 100% at the most recent analysis. During this time the metastases have remained stable.

**Figure 5:**
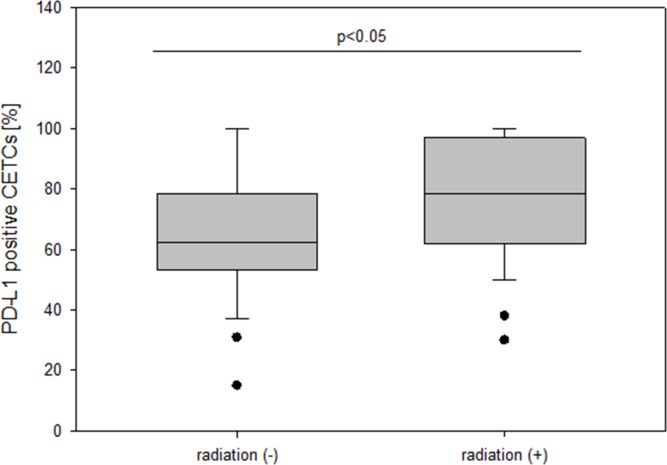
The frequency of PD-L1 positive CETCs (%) in breast cancer patients with and without radiation

30 (41.6%) patients received adjuvant radiotherapy, of which 20 patients were irradiated with standard regime (50 Gy in 25 fractions + 16 Gy boost). 10 patients older than 60 years obtained hypofractionated regime with 42.56 Gy in 16 fractions. Patients after radiotherapy (n=30) had a higher fraction of PD-L1 positive CETCs as compared to patients without radiotherapy (n=31) (median 77.4% vs 62.5%; p<0.05), regardless of radiation regime (Figure 5). Since inflammation occurring during radiation may stimulate PD-L1 expression on tumor cells we analysed CETCs from patients with a recent history of irradiation and observed the same phenomenon in our study.

We did not find any correlations between the numbers of PD-L1 positive CETCs and clinicopathological parameters in prostate, colorectal and lung cancer patients.

**Figure 6:**
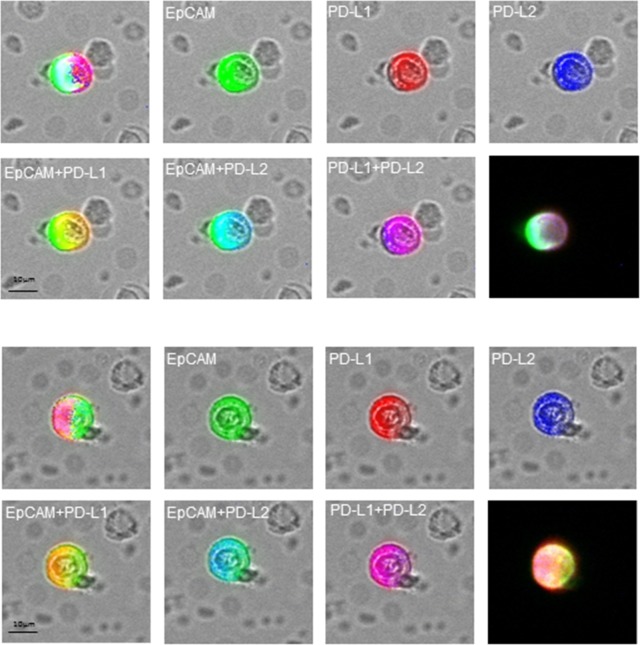
Fluorescence co-localization of EpCAM (green), PD-L1 (red) and PD-L2 (blue) on the CETCs in two representative results

**Figure 7:**
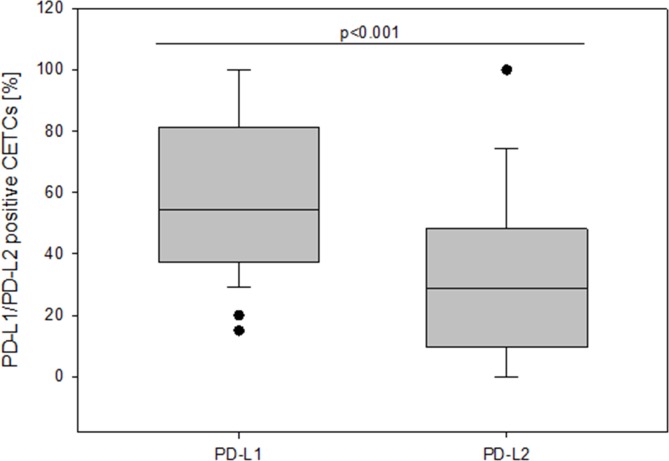
The frequency of PD-L1 and PD-L2 positive CETCs (%) in breast cancer patients

**Figure 8:**
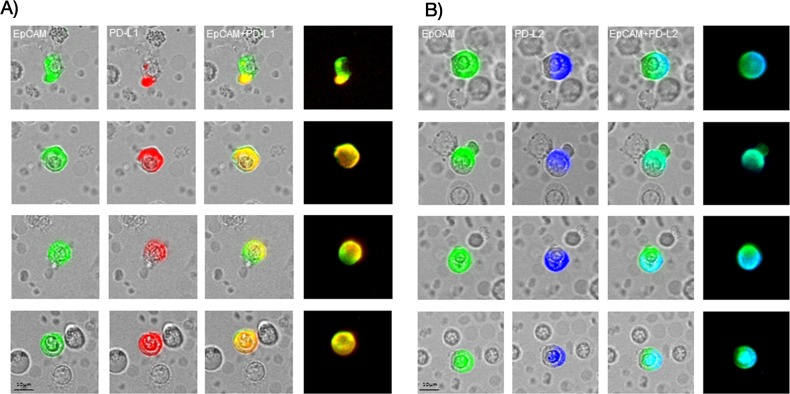
Illustrative CETCs pictures of double antibody staining for a) EpCAM (green) and PD-L1 (red) and b) EpCAM (green) and PD-L2 (blue) The expression of PD-L1 and PD-L2 is very heterogeneous and intensity of fluorescence varies strongly across CETCs from the same patients at one time point.

We, then, evaluated and compared the percentage of PD-L1 and PD-L2 positive CETCs in 28 breast cancer patients by performing co-expression analysis. The co-expression of PD-L1 and PD-L2 was confirmed in 82.1% of patients (Figure 6). In comparison with PD-L1, the percentage of PD-L2 positive CETCs was significantly lower (median PD-L1 54.6% vs median PD-L2 28.7%; p<0.001) (Figure 7) and did not correlate with any clinicopathological parameters. We found a substantial heterogeneity in PD-L1 and PD-L2 expression levels across the CETCs from the same patient at one time point (Figure 8a, 8b).

**Figure 9:**
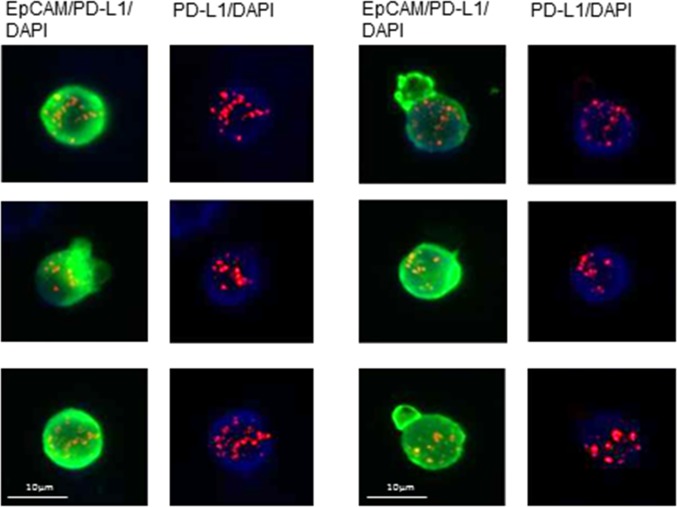
Illustrative examples of fluorescence in situ hybridization (FISH) for PD-L1 in CETCs from breast cancer patients

**Figure 10:**
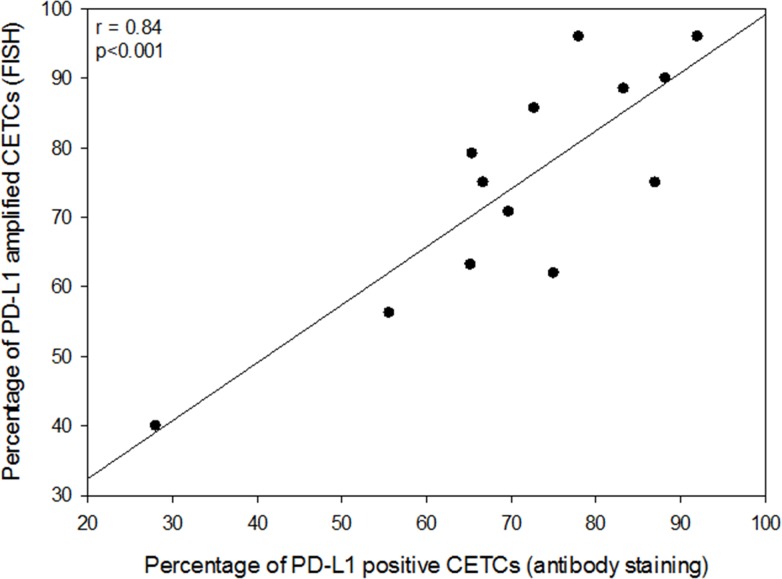
Correlation between the frequency of PD-L1 positive CETCs (antibody staining) and the frequency of PD-L1 amplified CETCs (FISH)

We next evaluated and compared the degree of concordance between PD-L1 expression and copy numbers in CETCs from the same patients in 13 breast cancer cases. PD-L1 amplified CETCs were detected in all examined cases (Figure 9). The percentage of amplified CETCs ranged from 62 to 96% with median 75% and was significantly associated with PD-L1 expression (r=0.84, P<0.001) in the examined patients (Figure 10).

## DISCUSSION

The PD-1/PD-L1 axis is a key suppressor of the cytotoxic immune response permitting cancer progression and metastasis and blockade of this pathway is a new promising therapeutic approach in oncology [13]. Clinical trials testing anti-PD-1 or anti-PD-L1 drugs have shown promising results with durable responses in different cancers [14]. Attention is now focused on the identification of a predictive biomarker to select patients who will actually benefit from a PD-1/PD-L1 blockade.

As there is no standard immunohistochemical technique, reports about the frequency of PD-L1 positivity in formalin fixed paraffin embedded (FFPE) breast cancer tissue sections varies widely in the literature. Ghebeh et al. [19] and Muenst et al. [20] reported very similar results with PD-L1 positivity in 34% and 23.4% of patients, respectively. A very recent study analyzing 192 specimens showed that PD-L1 expression was present in 56.6% of breast cancer cases [21]. In contrast, Ali et al. analyzed 3916 breast tumors and found that PD-L1 was expressed in only 1.7% of the total cases [22]. These differences may be due to different methods and antibodies applied and to the fact, that surface antigens often are altered or destroyed by the fixation procedures [23, 24].

In contrast, circulating tumor cells, which are the precursors of metastatic disease, are accessible and can be detected in a comparable way as other blood cells [25].

Tumor cells that are invasive and able to effectively evade cytotoxic T cells would have a greater selective advantage and likely contribute more to the progression of cancer disease. Very little data is available for PD-L1 expression on these cells and its significance in circulating tumor cells. For this reason we investigated PD-L1 expression on circulating epithelial tumor cells in breast, prostate, colorectal and lung cancer patients.

For the establishment of the PD-L1 assay on CETCs we tested different cancer cell lines (MCF-7, Sk-Br-3, SW620 and H820) and different clones of PD-L1 antibodies (clone 29E.2A3, clone 130021 and clone MIH1). Clone 29E.2A3 reacted positively only with H820 cells. In contrast to Mazel et al, we found no specific PD-L1 staining with clone 130021 neither with the H820 nor with the Sk-Br-3 cell line even after IFNγ treatment. However, with clone 29E.2A3 the most specific and unambiguous staining was observed in the H820 but not in the Sk-Br-3 cell line. The latter showed minimal binding in two other studies [26, 27]. Therefore we selected the H820 cell line as positive control and clone 29E.2A3 for the current study.

We found neither an association between the number of CETCs and different cancer types nor between the fraction of PD-L1 positive CETCs and different cancer types. In our approach 94.6% of breast cancer patients had PD-L1 positive CETCs although in different proportions. Mazel et al. using the CellSearch system found PD-L1 positive circulating tumor cells in 11/16 metastatic breast cancer patients (68.8%). The fraction of PD-L1 positive circulating tumor cells varied from 0.2-100% in individual patients [27], which is consistent with our results (PD-L1 positive CETCs ranged from 0 to 100%). However, in contrast to the results of Mazel et al. we were able to detect PD-L1 positive CETCs also in patients without metastases. This may allow treatment decisions already in the adjuvant situation [28]. The frequency of PD-L1 positive patients and the expression on CETCs was high as compared to results from tumor tissue. Apart from the better accessibility of surface antigens in circulating cells (as depicted above) a possible explanation for this discrepancy is the fact that tumor cells circulating in the blood are continuously in contact with T-lymphocytes. Upon tumor antigen recognition T cells produce interferon gamma, which through the interferon gamma receptor leads to beneficial antitumor effects, such as increased antigen presentation, increased production of chemokines and direct tumor growth arrest and apoptosis. However, interferon gamma pathway also leads to an adaptive increase in PD-L1 expression on the tumor cells resulting in escape of T cell cytotoxic effects [29]. This may be a reason why the frequency of PD-L1 positive CETCs in our analyses is significantly higher compared to tumor tissue. So far the discordance between PD-L1 status on CETCs and corresponding tumor tissue was not investigated but will be important for future assessment. Intratumoral heterogeneity, small sample size, lack of standardization and the fact that PD-L1 up-regulation is a dynamic biomarker might limit the interpretation of solid tumor biopsies and could lead to false negative results depriving patients from treatment that might benefit them. Additionally, other factors like cancer type, stage of cancer analyzed and treatment history can influence the results [13, 16, 30]. Furthermore, repeatable tissue biopsies are not feasible because it is an invasive, technically challenging procedure carrying risks to the patient. In contrast, liquid biopsy through the accessible and fairly non-invasive approach might allow for a dynamic characterization of PD-L1 expression on CETCs and serial monitoring the response to treatment [31, 32]. We, here, show that as a sign of successful immunotherapy, the total number of CETCs declined and the fraction of PD-L1 positive CETCs was significantly reduced. After discontinuation of checkpoint inhibitors the percentage of PD-L1 positive CETCs increased continuously and has achieved 100%. Taken together, immune-checkpoint inhibitors were able to eliminate PD-L1 positive CETCs from the peripheral blood of this breast cancer patient. It has been shown that persistence of PD-L1 positive circulating tumor cells correlates with poor prognosis and might reflect a mechanism of therapy escape [30]. Results regarding PD-L1 expression in tumor tissue and overall survival (OS) or disease free survival (DFS) are contradictory and the status of PD-L1 can either correlate with poor prognosis, better prognosis or show no correlation with prognosis at all. Muenst et al. postulated that PD-L1 expression is a negative prognostic factor of poor outcomes in breast cancer [20]. In contrast, Reiss et al. suggested that PD-L1 could be a good prognostic biomarker for OS in breast cancer [33]. In our study patients with metastatic disease had higher numbers of PD-L1 positive CETCs compared to patients without distant metastasis. Baptista et al. noticed that PD-L1 expression in tumor samples was significantly correlated with recurrence at distant sites [21]. Here we show that the number of PD-L1 positive CETCs correlates with the aggressiveness of tumor.

In the adjuvant situation chemo- and radiotherapy are the major components of cancer treatment but many patients get local recurrence or metastasis. The association of PD-L1 expression and obtaining radiotherapy found in our study correlates well with the known inflammatory effect of radiotherapy. Dovedi et al. showed that fractionated radiotherapy is responsible for an increased IFNγ production by CD8^+^ T cells mediating up-regulation of PD-L1 expression on tumor cells. Additionally, there is a strong correlation between PD-L1 expression on tumor cells and lymphocytic infiltration not only among tumors but also within regional sites in a tumor [13, 34, 35].

We were surprised to observe that 100% of prostate cancer patients had PD-L1 positive CETCs. Also in prostate tumor tissue PD-L1 expression seems to be elevated in comparison to other tumor types. Gevensleben et al. detected PD-L1 expression in 61.7% of primary prostate cancers [36]. Massari and colleagues recent study showed that PD-L1 was expressed in 50% of castration-resistant prostate adenocarcinoma [37].

The results reporting PD-L1 expression in colorectal cancer are highly diverse. Whereas Masugi et al. [38] found that 89% of colorectal carcinomas exhibited high tumor PD-L1 expression Lee et al. and Rosenbaum et al. reported on low levels of PD-L1 expression in colorectal cancer (5% and 9%, respectively) [39, 40] again possibly due to differences in methods applied. The frequency of patients with PD-L1 positive CETCs in our approach was 94.5% and thus fits rather to the former results of Masugi et al.

Also in NSCLC reports on PD-L1 expression varies highly ranging from 7.4% to 72.7% [41]. With respect to circulating tumor cells in patients with advanced NSCLC Nicolazzo et al. found that 95% of patients had a subpopulation of PD-L1 positive of PD-L1 positive [31] similar to our results of 82% in NSCLC patients.

Detection of copy number variants which, differently to the difficulties in antibody-dependent approaches, is independent of most fixation procedures can be an alternative method to IHC. The PD-L1 gene is located on chromosome 9p24.1 and the amplification of this gene locus has been reported in lymphomas [42], triple negative breast cancer [43] and NSCLC [44]. The up-regulation of proteins may be due to an increase in copy numbers and the overexpression of PD-L1 is frequently observed in PD-L1 amplified cases in such tumors. Information on the PD-L1 copy number status was lacking in CETCs so far. Here, we showed for the first time that PD-L1 copy numbers were increased and PD-L1 copy number gains were associated with PD-L1 expression on CETCs.

The role of PD-L2 in evading the immune system is not fully understood. Comparing the expression level of both PD-1 ligands on CETCs in breast cancer patients we observed that frequency of cells with PD-L2 expression was significantly lower compared to the frequency of PD-L1 expression. Therefore PD-L2 may play only a marginal role in immunotherapy. This is in agreement to previous studies which found that in comparison to PD-L1, PD-L2 expression was observed less frequently in tissue samples. PD-L2 expression may be relatively restricted to macrophages, dendritic cells, and fibroblasts [12, 44]. To the best of our knowledge this is the first report on PD-L2 determination on circulating tumor cells.

Breakthrough therapy with checkpoint inhibitors in the treatment of cancer may gain even more importance in the near future. Drugs inhibiting PD-L1 and PD-1 exhibit a favorable toxicity profile, but so far treatment is applied only to a subset of patients. There is a need to identify reliable biomarkers to predict response to these therapies and to facilitate patient selection [18]. Taken together, the high frequency of PD-1 ligand expression by circulating epithelial tumor cells provides an important rationale for the capacity of antibody blockade of this pathway already in the adjuvant situation to enhance immune response. Furthermore, analysis of CETCs for PD-L1 expression could be useful for therapy stratification and monitoring response to therapy. Additionally, during course of therapy serial tests could allow the detection of early resistance development.

## MATERIALS AND METHODS

### Blood collection and processing

Peripheral blood (7.5 ml) from altogether 128 patients with breast (72/56%), prostate (27/21%), colorectal (18/14%) and lung (11/9%) cancer in different stages of disease was drawn into normal blood count tubes with ethylenediaminetetraacetic acid (EDTA) as an anticoagulant and processed within 48 hours of collection. In parallel, healthy control blood samples were collected from 25 female and male donors aged from 20-40 years. In patients with primary breast cancer (n=55) the sampling of peripheral blood was carried-out 6-12 weeks after end of standard therapy (tumor resection, adjuvant chemotherapy, adjuvant radiotherapy). In patients with local or distant recurrence the blood was collected prior to treatment of recurrent disease.

### maintrac^®^

For CETC enumeration and further characterization the maintrac^®^ approach was used, as reported previously [5]. Briefly, 1 ml blood was subjected to red blood cell lysis using 15 ml of erythrocyte lysis solution (Qiagen, Hilden, Germany) for 15 min in the cold, spun down at 700 g and re-diluted in 500 μl of PBS-EDTA. 5μl of fluorescein-isothiocyanate (FITC)-conjugated anti-human epithelial cell adhesion molecule antibody (EpCAM) (clone HEA-125, Miltenyi Biotec GmbH, Germany) at a final concentration of up to 10^7^ cells/100 μl cell suspension were added and incubated for 15 min in cold. The corresponding isotypic control for EpCAM (Mouse IgG1_K_ FITC, Miltenyi Biotec GmbH, Germany) was used at the same final concentration. The samples were subsequently diluted with 430 μl PBS-EDTA. A defined volume of the cell suspension and propidium iodide (PI) (Sigma-Aldrich, USA) was transferred to wells of ELISA plates (Greiner Bio-one, USA). Analysis of red and green fluorescence of the cells was performed using a Fluorescence Scanning Microscope, ScanR, (Olympus, Hamburg, Germany), enabling detection and relocation of cells for visual examination of EpCAM positive cells. For data analysis we used the ScanR Analysis software (Olympus, Hamburg, Germany). Vital CETCs were defined as EpCAM-positive cells, lacking in CD45-/ PI-staining and with intact morphology, and only these cells were counted. We used fluorospheres (Flow-Check 770, Beckman Coulter) for daily verification of optical components and detectors of the microscope, which are required to ensure the consistent analysis of samples.

### Cell lines

H820 lung cancer cells which were used as a positive control for PD-L1 analysis were obtained from the American Type Culture Condition (ATCC, Manassas, USA). Three different clones of anti-PD-L1 antibodies were tested: (1) clone 29E.2A3 (BioLegend, San Diego, USA), (2) clone 130021 (R&D Systems, Minneapolis, USA), (3) clone MIH1 (eBioscience, San Diego, USA) (Figure 1). SW620 colorectal cancer cells, MCF7 and Sk-Br-3 breast cancer cells were used as a negative control and were obtained from the CLS cell lines service (Eppenheim, Germany) (Figure 2). H820 cells were grown in RPMI-1640 medium with 5% fetal bovine serum (FBS, Gibco, Thermo Fisher Scientific, Waltham, USA), SW620 and Sk-Br-3 cells were grown in Dulbecco's modified Eagle's medium with 4,5g/L glucose, 2mM L-glutamine (Gibco, Thermo Fisher Scientific, Waltham, USA) and 10% FBS. Cells were maintained at 37°C in 5% CO_2_. MCF-7 cells were grown in Minimum Essential Eagle ready-to-use medium (CLS cell lines service (Eppenheim, Germany). For immunofluorescence analysis cells were detached from cell culture flasks using StemPro^®^ Accutase^®^ Cell Dissociation Reagent (Gibco, Thermo Fisher Scientific, Waltham, USA) washed and stained for PD-L1 with the same protocol like a patient sample.

### PD-L1/−2 analysis

**Figure 11:**
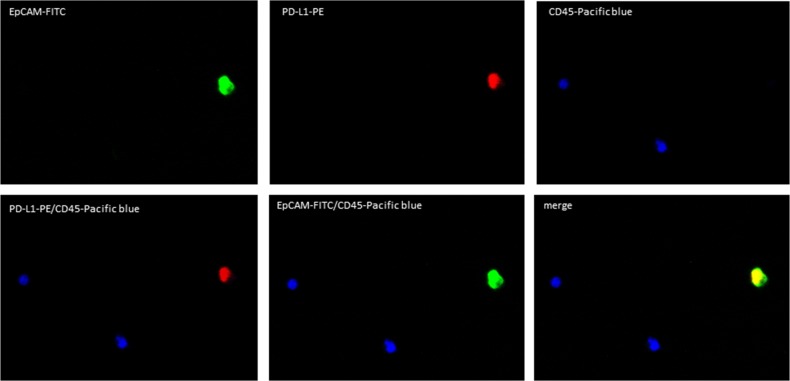
Fluorescence microscope images of PD-L1 positive CETCs CETC is positive for EpCAM and PD-L1 and strictly negative for CD45.

**Figure 12:**
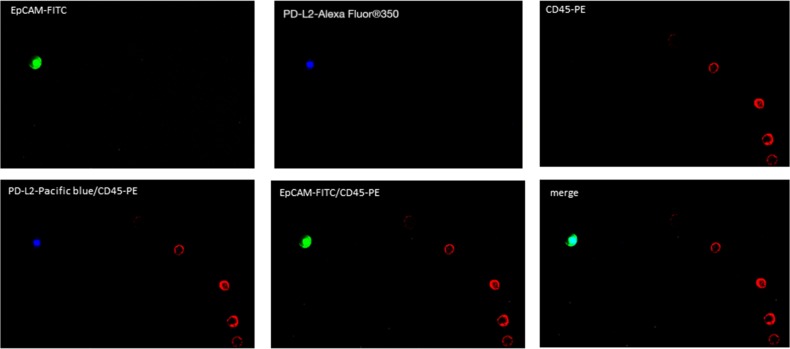
Fluorescence microscope images of PD-L2 positive CETCs CETC is positive for EpCAM and PD-L2 and strictly negative for CD45.

The analyses of PD-L1 and PD-L2 expression on the CETCs were performed with an extended maintrac^®^ approach. For PD-L1 expression analysis we used an anti-human PD-L1 phycoerythrin (PE)-conjugated antibody (clone 29E.2A3, BioLegend, San Diego, USA) at a final concentration of 0.2 μg/ml and for PD-L2 we used an anti-human PD-L2 Alexa Fluor^®^ 350 conjugated antibody (clone 176611, novus biologicals, Littleton, USA) at a final concentration of 2 μg/ml. The corresponding isotypic controls for PD-L1 (Mouse IgG2b PE, BioLegend, San Diego, USA) and PD-L2 (Mouse IgG2b Alexa Fluor^®^ 350, Novus biologicals, Littleton, USA) were used at the same final concentration. Finally, cells were visually inspected looking for a green, red and blue surface staining, but also a well-preserved nucleus (Figure 8a, 8b). For excluding expression of PD-L1/PD-L2 on hematopoetic cells we additionally performed staining with EpCAM-FITC, PD-L1-PE/PD-L2-Alexa Fluor® 350 and CD45-Pacific blue/CD45-PE antibodies (Figure 11, 12). The results for PD-L1 and PD-L2 were calculated as percentage of total number of CETCs.

### Fluorescence-in-situ-hybridization (FISH)

For FISH analyses the *PD-L1* gene was tested by using a dual fluorescence kit (CD274(PD-L1)/CEN9q FISH Probe, abnova, Taiwan) containing the *CD274* (*PD-L1)* gene (9p24, directly labeled with Texas Red) and *CEN9q* (9q21, labeled with FITC). Patient cells were transferred onto Poly-L-Lysin coated slides. Before hybridization slides were fixed with 4% paraformaldehyde for 10 min and treated for 10 min with proteinase K at room temperature. In a next step, cells were denatured for 5 min at 72°C in 70% formamide - 2 x standard saline citrate solution, air dried and dehydrated in 70%, 85% and 96% ethanol. After overnight hybridization at 37°C in a humidified chamber, slides were washed, air dried and counterstained with 0.2 μM DAPI in an anti-fade solution. At least 20 nuclei per sample were counted. CETCs were positive for PD-L1 amplification when more than 3 PD-L1 signals in one cell were counted. The final results for PD-L1 amplification were calculated as percentage of 20-30 visually expected EpCAM positive cells.
